# Altering the coenzyme preference of xylose reductase to favor utilization of NADH enhances ethanol yield from xylose in a metabolically engineered strain of *Saccharomyces cerevisiae*

**DOI:** 10.1186/1475-2859-7-9

**Published:** 2008-03-17

**Authors:** Barbara Petschacher, Bernd Nidetzky

**Affiliations:** 1 Institute of Biotechnology and Biochemical Engineering, Graz University of Technology, Petersgasse 12/I, A-8010 Graz, Austria

## Abstract

**Background:**

Metabolic engineering of *Saccharomyces cerevisiae *for xylose fermentation into fuel ethanol has oftentimes relied on insertion of a heterologous pathway that consists of xylose reductase (XR) and xylitol dehydrogenase (XDH) and brings about isomerization of xylose into xylulose via xylitol. Incomplete recycling of redox cosubstrates in the catalytic steps of the NADPH-preferring XR and the NAD^+^-dependent XDH results in formation of xylitol by-product and hence in lowering of the overall yield of ethanol on xylose. Structure-guided site-directed mutagenesis was previously employed to change the coenzyme preference of *Candida tenuis *XR about 170-fold from NADPH in the wild-type to NADH in a Lys^274^→Arg Asn^276^→Asp double mutant which in spite of the structural modifications introduced had retained the original catalytic efficiency for reduction of xylose by NADH. This work was carried out to assess physiological consequences in xylose-fermenting *S. cerevisiae *resulting from a well defined alteration of XR cosubstrate specificity.

**Results:**

An isogenic pair of yeast strains was derived from *S. cerevisiae *Cen.PK 113-7D through chromosomal integration of a three-gene cassette that carried a single copy for *C. tenuis *XR in wild-type or double mutant form, XDH from *Galactocandida mastotermitis*, and the endogenous xylulose kinase (XK). Overexpression of each gene was under control of the constitutive TDH3 promoter. Measurement of intracellular levels of XR, XDH, and XK activities confirmed the expected phenotypes. The strain harboring the XR double mutant showed 42% enhanced ethanol yield (0.34 g/g) compared to the reference strain harboring wild-type XR during anaerobic bioreactor conversions of xylose (20 g/L). Likewise, the yields of xylitol (0.19 g/g) and glycerol (0.02 g/g) were decreased 52% and 57% respectively in the XR mutant strain. The xylose uptake rate per gram of cell dry weight was identical (0.07 ± 0.02 h^-1^) in both strains.

**Conclusion:**

Integration of enzyme and strain engineering to enhance utilization of NADH in the XR-catalyzed conversion of xylose results in notably improved fermentation capabilities of recombinant *S. cerevisiae*.

## Background

**Figure 1:**
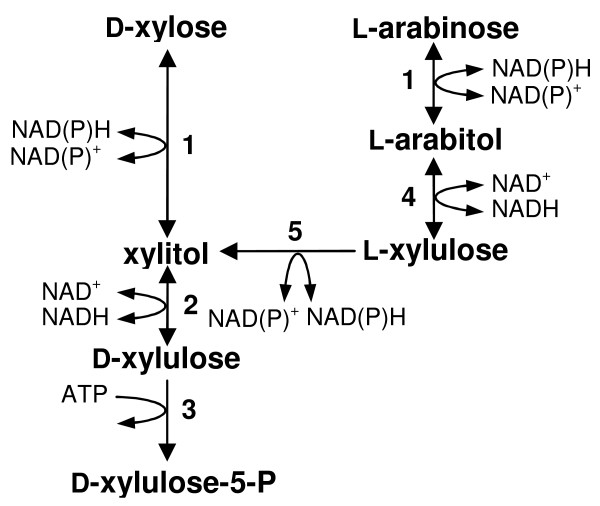
**Pathways for utilization of D-xylose and L-arabinose in fungi**. 1, aldose reductase (EC 1.1.1.21); 2, xylitol dehydrogenase (EC 1.1.1.9); 3, xylulose kinase (EC 2.7.1.17); 4, L-arabinitol 4-dehydrogenase (EC 1.1.1.12); 5, L-xylulose reductase (EC 1.1.1.10).

Rising oil prices and a growing awareness of a possible climate change caused by greenhouse gas emission have recently led to rekindled interest in bioethanol as a CO_2_-neutral liquid fuel. Lignocellulose will be the prime choice of feedstock for the production of bioethanol if major technical problems in its conversion can be overcome [1,2]. One notable difficulty has been in the development of robust microbial strains capable of fermenting efficiently all types of sugars present in the cellulose and hemicellulose fractions of the raw material [3-6]. While *Saccharomyces cerevisiae *is a top candidate to be used in the fermentation of D-glucose and the hemicellulose-derived hexoses D-galactose and D-mannose, the organism in its wild-type form cannot utilize the pentoses D-xylose and L-arabinose [3,4,6-11] which constitute ≥ 80% of the total sugar contained in typical hemicellulose hydrolyzates [12]. The particular deficiency of *S. cerevisiae *is caused by an insufficient expression of pathways which in other yeasts deliver either of the two sugars as D-xylulose 5-phosphate into the central metabolism (Figure 1).

Initial efforts of strain engineering in *S. cerevisiae *targeted expansion of the substrate spectrum towards D-xylose and involved heterologous expression of *Pichia stipitis *genes encoding xylose reductase (XR) and xylitol dehydrogenase (XDH) [13-15]. Overexpression of an endogenous xylulose kinase (XK) gene was used to eliminate a putative kinetic bottleneck in the phosphorylation of D-xylulose [16-18]. Ethanol yields obtained with this first generation of xylose-fermenting strains were far below the theoretical maximum of 0.51 g/g xylose, because a large part of the xylose consumed was excreted as xylitol. This was attributed to an imbalanced coenzyme utilization in the steps catalyzed by a dual specific, NADPH-preferring XR and a strictly NAD^+^-dependent XDH [reviewed in [1-6,10]], a fundamental problem recognized before in seminal studies of xylose utilization by yeasts [13,19-21]. Strategies to decrease xylitol formation have included re-oxidation of excess NADH by external electron acceptors [22], manipulations of the yeast central metabolism at various NADP(H) or NAD(H)-dependent steps outside the xylose pathway [23-27] and more recently, engineering of the coenzyme specificity of XR [28,29] or XDH [30-32]. The conceptually most compelling approach is replacement of XR and XDH by a xylose isomerase (XI), so that xylose can be directly transformed into xylulose [33,34]. However, identification of a candidate XI for high-level functional expression in *S. cerevisiae *proved difficult [35]. Pronk and coworkers isolated a novel XI from a fungal source (*Pyromyces *sp. ATCC 76762) [32] and succeeded in constructing a xylose-fermenting yeast strain based on this enzyme [36-38]. High ethanol yields of 0.42 g/g and almost no xylitol formation were observed in batch fermentations on 20 g/L xylose [36]. A recent comparison of xylose-fermenting yeast strains carrying the *P. stipitis *XR-XDH pathway or the *Pyromyces *XI pathway revealed that the XR-XDH strategy resulted in a 2.6-fold faster ethanol production rate although the overall ethanol yield (0.33 g/g) was significantly lower than in the strain carrying XI (0.43 g/g) [39]. An engineered XR-XDH pathway in which formation of excess NADH is reduced while the fluxional efficiency of the wild-type pathway is retained could therefore be the key to the construction of new xylose-fermenting strains that combine both good yield and productivity.

We have recently employed structure-guided site-directed mutagenesis to change the coenzyme preference of *Candida tenuis *XR from NADPH (33-fold) in the wild-type enzyme to NADH (5-fold) in a Lys^274^→Arg Asn^276^→Asp double mutant (K274R-N276D) [40]. According to its kinetic parameters, K274R-N276D is expected to fully substitute for the wild-type enzyme during NADH-dependent reduction of xylose in a recombinant strain of *S. cerevisiae*. Results of *in vitro *assays suggest that the double mutant will probably show indiscriminate usage of NADH and NADPH under physiological reaction conditions [41]. This work was carried out to verify the predicted *in vivo *function of K274R-N276D and analyze consequences in xylose-fermenting *S. cerevisiae *that result from a well defined change in XR coenzyme specificity.

## Results

### Development of stable xylose-fermenting strains of *S. cerevisiae *expressing *C. tenuis *XR in wild-type or K274R-N276D double mutant form

Two isogenic yeast strains were derived from the laboratory strain *S. cerevisiae *CEN.PK 113-7D. Single copies for the genes encoding *Ct*XR in wild-type (strain BP000) or K274R-N276D double mutant form (strain BP10001) and XDH from *Galactocandida mastotermitis *(*Gm*XDH) were inserted in the URA3-locus of the yeast genome together with an extra copy of the endogenous XK gene (Table 1). Both strains grew aerobically on xylose with nearly identical specific growth rates of about 0.07 h^-1^. Their specific rates of growth and substrate consumption during aerobic and anaerobic conversion of glucose were also very similar. As expected, the reference strain *S. cerevisiae *CEN.PK 113-7D was unable to utilize xylose for aerobic growth or as fermentation substrate (data not shown). Table 2 summarizes specific activities of XR, XDH, and XK in cell extracts of BP000 and BP10001 cultivated aerobically in mineral medium containing 20 g/L of each glucose and xylose. However, xylose could be omitted from the medium without any effect on the specific activity of each of the three enzymes. For reasons we do not understand, BP10001 showed a significantly, about 1.8-fold higher level of XR activity than BP000, irrespective of various growth conditions and times of cell harvest used. The specific activity of XK was also higher (≈ 40%) in BP10001 than in BP000. The levels of XDH activity were comparable in the two strains. Cell extracts prepared by using Y-PER contained 1.4-fold higher specific XR activity than others obtained by disrupting exactly comparable cell material in a French Press. While the result is explicable on account of XR activity enhancement by the detergents present in Y-PER (data not shown; see ref. [42] for the effect of non-ionic detergents on activity and stability of *Ct*XR), it also provides a note of caution regarding the comparison of enzyme activities that are based on different methods of yeast cell disruption.

**Table 1:** Relevant genotypes and phenotypes of strains BP000 and BP10001

**Strain**	**Relevant genotype**	**Phenotype**
BP000	CEN.PK 113-5D ura3::(GPDp-XKS1-CYC1t, GPDp-*Ct*XRWt-CYC1t, GPDp-*Gm*XDH-CYC1t)	Produces *Ct*XR wild type + *Gm*XDH, overexpresses XKS1
BP10001	CEN.PK 113-5D ura3::(GPDp-XKS1-CYC1t, GPDp-*Ct*XRDm-CYC1t, GPDp-*Gm*XDH-CYC1t)	Produces *C*tXR K274RN276D double mutant + *Gm*XDH, overexpresses XKS1

GPDp and CYC1t stand for the *S. cerevisiae *TDH3 promoter and CYC1 terminator, respectively.

**Table 2:** XR, XDH and XK activities in crude cell extracts of BP000 and BP10001. Cells were grown aerobically on a mixed sugar substrate containing 20 g/L of each glucose and xylose and were then disrupted with Y-Per.

**Strain**	**XR activity U/mg^a^**		**XDH activity U/mg**	**XK activity U/mg**
BP000	NADH	0.15 ± 0.01^b^	1.1 ± 0.1	1.7 ± 0.2
	NADPH	0.18 ± 0.01		
BP10001	NADH	0.26 ± 0.01	1.3 ± 0.1	2.4 ± 0.1
	NADPH	0.33 ± 0.01		
Cen.PK 113-7D	NADH	n.d.^c^	n.d.	0.14 ± 0.04
	NADPH	0.008 ± 0.003		

^a ^Activities measured using a coenzyme concentration of 350 μM and a xylose concentration of 700 mM

^b ^Mean values ± S.D. from three independent experiments including cultivation and disruption of cells and measurement of protein and activity

^c ^n.d. not detectable.

**Figure 2:**
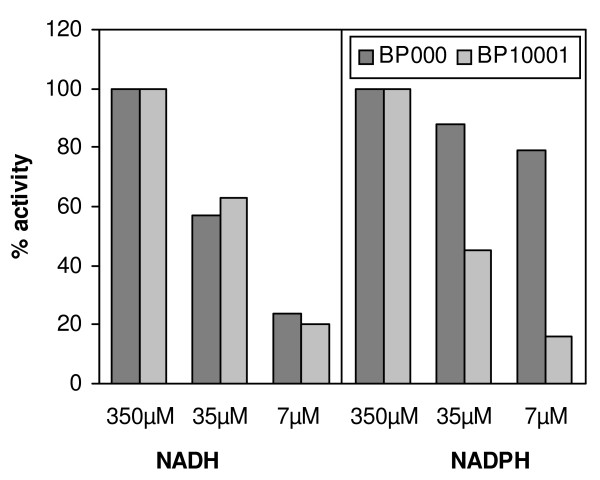
**Comparison of XR activities of BP000 and BP10001 at different cofactor concentrations**. Cells were grown aerobically on 20 g/L glucose and 20 g/L xylose and disrupted with Y-Per reagent. One hundred % specific activity of strain BP000 corresponds to values of 0.15 U/mg with NADH and 0.18 U/mg with NADPH. In strain BP10001, the specific activities are 0.26 U/mg with NADH and 0.33 U/mg with NADPH.

Figure 2 compares XR activities of BP000 and BP10001 recorded at three concentrations of NADH and NADPH. The relative decrease in enzymatic rate in response to lowering the level of NADH from 350 μM to 7 μM was similar in the two strains. By contrast, the drop in activity caused by the same change in the concentration of NADPH was much more significant in BP10001 than in BP000. These results are in excellent agreement with expectations from the *K*_m _values of purified wild-type (NADH: 38 μM; NADPH: 3 μM) and K274R-N276D (NADH: 41 μM; NADPH: 128 μM) [40]. They also serve to verify functional expression of the double mutant in BP10001.

### Oxygen-limited conversion of xylose in shake-flask cultures

**Figure 3:**
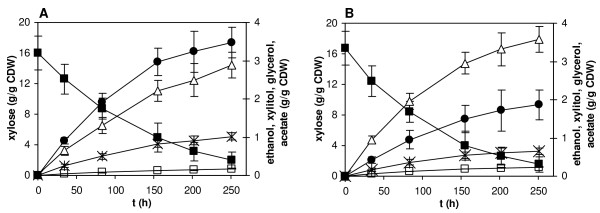
**Xylose utilization and product formation during oxygen-limited shake flask cultivation of BP000 (panel A) and BP10001 (panel B)**. Xylose (full squares), ethanol (triangles), xylitol (circles), glycerol (stars) and acetate (empty squares) were analyzed by HPLC. The biomass concentration was constant at 1.4 ± 0.1 g/L for BP000 and 1.5 ± 0.1 g/L for BP10001. Error bars show the S.D. of triplicate fermentation experiments.

Batch conversions of xylose by glucose-grown resting cells of BP000 and BP10001 were carried out under oxygen-limited reaction conditions ([O_2_] ≤ 20 μM) in shake flasks using a mineral medium that contained 20 g/L sugar. Typical fermentation time courses are shown in Figure 3 and parameters derived from their analysis are summarized in Table 3. No biomass was formed under these conditions. In a carbon balance calculated from the data in Figure 3 whereby CO_2 _was inferred from the ethanol and acetate values, only ≤ 7% of the carbon from xylose remained unaccounted for. In comparison with the reference strain BP000, the XR double mutant strain BP10001 showed 40% enhanced ethanol yield. Its production of xylitol and glycerol was decreased by 53% and 30%, respectively. The yield of acetate was generally low in both strains, however, enhanced by about 50% in BP10001.

**Table 3:** Comparison of xylose fermentation by the recombinant *S. cerevisiae *strains BP000 and BP10001

	**BP000**		**BP10001**	
	Shake flask (oxygen limited)	Bioreactor (anaerobic)	Shake flask (oxygen limited)	Bioreactor (anaerobic)

***q*_xylose_^a^**	0.07	0.06	0.07	0.08 ^d^
***Y*_ethanol_^b^**	0.24	0.24	0.34	0.34
***Y*_xylitol_**	0.35	0.39	0.17	0.19
***Y*_glycerol_**	0.091	0.048	0.063	0.021
***Y*_acetate_**	0.019	0.019	0.031	0.020
**C-recovery**	93%^c^	101%	94%^c^	96%

^a ^Xylose uptake rates (*q*) are given in g/g CDW/h

^b ^Yields (*Y*) are given in g/g xylose.

^c ^For the calculation of the carbon balance, it was assumed that one mole of CO_2 _was formed per mol of ethanol or acetate

The given values were obtained by analyzing samples taken in the range 45 – 60% xylose consumption. Shake flask experiments were done in triplicates, bioreactor experiments in duplicate. Mean values are shown. Their relative S.D. was < 5% with the exception of values of *q*_xylose _and *Y*_acetate _from shake flasks experiments whose S.D. was ≤ 14%. ^d ^he S.D. for this value was about 30%.

### Anaerobic conversions of xylose in bioreactor experiments

**Figure 4:**
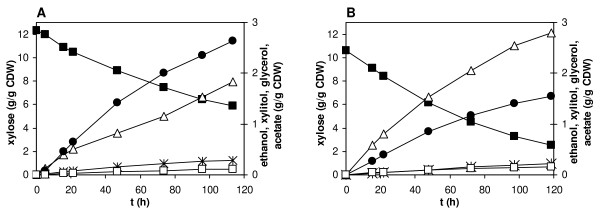
**Xylose utilization and product formation during anaerobic bioreactor cultivation of BP000 (panel A) and BP10001 (panel B)**. Xylose (full squares), ethanol (triangles), xylitol (circles), glycerol (stars) and acetate (empty squares) were analyzed by HPLC. The biomass concentration was constant at 1.6 ± 0.1 g/L for BP000 and 1.8 ± 0.1 g/L for BP10001.

To verify the results of shake flask experiments under well controlled fermentation conditions where in particular the concentration of dissolved oxygen was monitored continuously, we compared anaerobic conversions of xylose (20 g/L) by BP10001 and BP000 carried out in a Braun Biostat bioreactor. Results are summarized in Figure 4 and Table 3. For both strains, the physiological parameters measured in the bioreactor were in good agreement with the ones obtained in shake flask. The glycerol yield was an exception as bioreactor experiments gave significantly lower values in this case. Likewise, the acetate yield for strain BP10001 was lower in bioreactor compared to shake flask cultivations and identical to the corresponding acetate yield for the control strain (0.019 g/g). Therefore, positive effects in the XR double mutant strain on xylose fermentation in shake flask could be scaled up fully to the laboratory bioreactor.

## Discussion

### Protein engineering to improve coenzyme recycling in the metabolic steps catalyzed by XR and XDH

Biochemical constraints dictate that anaerobic conversion of xylose into ethanol is possible only when XR and XDH have matching coenzyme specificities [19,21,43]. The xylose pathway from *Pichia stipitis *which has served as point of departure for the construction of numerous xylose-fermenting strains of *S. cerevisiae *[4,6] does not fulfill this requirement well. Its XDH is strictly specific for NAD^+ ^[44] while the XR strongly prefers NADPH over NADH [45,46]. Using known natural enzymes, the assembly of a chimeric pathway in which XR and XDH show exactly comparable utilization of NADP(H) and NAD(H) appears to be currently out of reach.

Protein engineering has therefore been pursued to make the coenzyme specificity of XR or XDH better compatible with that of the corresponding partner enzyme of the xylose pathway. Following the early studies by Metzger and Hollenberg [47], Makino and coworkers succeeded in creating a NADP(H)-dependent version of *P. stipitis *XDH through rational design [32]. A notable feature of the best improved multiple mutant of XDH was a catalytic efficiency for the NADP^+^-dependent reaction that exceeded about 3.8-fold the corresponding efficiency of the wild-type using NAD^+^. While XDH from yeast and fungal sources is typically a Zn^2+^-dependent enzyme evolutionary related to medium-chain dehydrogenases/reductases [48,49], bacterial polyol dehydrogenases possessing activity with xylitol do not use an active-site metal in catalysis and are found with the short-chain dehydrogenase/reductase superfamily of proteins [50]. Ehrensberger and Wilson [51] determined a 1.9 Å crystal structure of XDH from *Gluconobacter oxidans *based on which they were able to convert the NAD^+^-dependent wild-type enzyme into a strictly NADP^+^-specific variant that had retained about 14% of the original catalytic efficiency for oxidation of xylitol.

Successful creation of a highly active XR mutant featuring a substantially lower preference for NADPH than the wild-type enzyme has strongly benefited from crystal structures of the enzyme from *C. tenuis *bound with NADP(H) [52] and NAD(H) [53]. Some of the mutations found to be useful in *Ct*XR [40] were later also introduced at homologous positions of the amino acid sequence of XR from *P. stipitis *[28,29,54] (see later). Selection of the K274R-N276D doubly mutated *Ct*XR for the *in vivo *experiments reported herein was based on a detailed steady-state kinetic characterization of a series of single and multiple-site variants of *Ct*XR [40] and included analysis of mixed coenzyme utilization in the presence of physiological concentrations of NADPH and NADH [41]. The double mutant eliminates the 33-fold preference of the wild-type for reaction with NADPH; however, it is clearly not a perfectly NADH-dependent enzyme. With the desired application for xylose fermentation in mind, it was important to also consider the possible effect of the mutated XR on the fluxional efficiency of the xylose pathway. The K274R-N276D double mutant was expected from its kinetic parameters to substitute the wild-type enzyme in the NAD(H)-dependent conversion of xylose without introducing an extra kinetic bottleneck.

### Metabolic consequences of altering the coenzyme preference of XR in a xylose-fermenting strain of *S. cerevisiae*

The discussion will focus on physiological effects observed in stable xylose-fermenting strains of *S. cerevisiae *where the relevant genes were integrated into the yeast genome. Note, however, that preliminary reports have been published in which xylose fermentation by yeast strains expressing mutated *P. stipitis *XR or XDH from multi-copy plasmid vectors was investigated. They support the general idea that enhanced recycling of NADH [28,29] or NADPH [30,31] in the XR-XDH pathway helps decreasing xylitol formation and can eventually increase the ethanol yield. However, two independent studies, in which exactly the same mutants of *P. stipitis *XDH were examined, reached opposite conclusions regarding the effect on ethanol yield resulting from the usage of NADP^+ ^instead of NAD^+ ^in the XDH step [30,31]. These results emphasize the possible ambiguity in tracing back changes in strain physiology to the modification of the cosubstrate specificity of XR or XDH.

Therefore, the relevant phenotypes of the two isogenic yeast strains constructed in this work were carefully analyzed. Gene expression under control of the TDH3 promoter yielded levels of specific activity for XR (utilizing NADH), XDH, and XK that were about half those obtained by other groups who used the phosphoglycerate kinase 1 promoter for expressing the genes of the *Pichia stipitis *xylose pathway along with the endogenous XK gene [17,55,56]. (The comparison is relevant because purified *Ct*XR [42] and *Gm*XDH [57] display similar specific activities as the corresponding *P. stipitis *enzymes [44-46].) The observed ratio of the specific activities of XR-NADH, XDH, and XK was 1: ≈5–7: ≈10 and lies within the window of operation recommended by Hahn-Hägerdal and co-workers [55,58].

We were concerned about the difference in specific XR and XK activities found in strains BP000 and BP10001 that was substantially larger than expected from the estimated experimental error of 15 – 20% for the entire procedure of cell disruption and activity measurement. A gene copy number effect can be ruled out considering that (1) chromosomal integration of the three overexpressed genes occurred in a single step; and (2) unlike XR and XK, the specific activity of XDH was identical in both strains. However, for the purpose of strain comparison for xylose fermentation it may be noted that the specific uptake rates for the xylose substrate were very similar in BP000 and BP10001. We therefore regarded the two yeast strains as a suitable system for examining metabolic consequences resulting from the change in XR coenzyme specificity. The unknown source of variation in the specific enzyme activities was not further pursued.

The 52% decrease in xylitol yield resulting from the genetic replacement of wild-type *Ct*XR by the K274R-N276D double mutant is quite significant in comparison to the success other metabolic engineering strategies have had in suppressing xylitol formation [for comprehensive reviews, see [1-5]], not only in terms of the magnitude of the effect but also because it was accompanied by similar changes in ethanol yield (42% increase) and glycerol yield (57% decrease). The acetate yield in bioreactor cultivations of the two strains was not affected within limits of the experimental error. Therefore, alteration of XR coenzyme specificity appears to have caused a global metabolic response, which contributes to a comprehensive improvement of the distribution of fermentation products.

It is interesting to bring into comparison these data with results of a detailed study by Jeppson et al. [56] who examined the effect of substituting wild-type XR from *P. stipitis *by a Lys^270^→Met mutant thereof, which according to studies of Lee and co-workers [59] exhibits a 17-fold higher *K*_m _for NADPH than the native enzyme. The analogous site-directed replacement in *Ct*XR, Lys^274^→Met, caused improvement of the coenzyme selectivity, NADPH compared to NADH, from a value of 33 in the wild-type enzyme to 5.5 in the mutant [40]. However, it was also accompanied by a more substantial, 20-fold decrease in catalytic efficiency for the NADH-dependent reduction of xylose [40].

Despite the expected strong impairment of XR physiological function resulting from the mutation Lys^270^→Met [59], the yeast strain harboring a single gene copy for *P. stipitis *mutant XR consumed xylose in batch fermentations as fast as the isogenic control strain that contained native XR, and it produced less xylitol (0.17 vs. 0.29; 42% decrease) and more ethanol (0.36 vs. 0.31; 16% increase) [56]. Formation of acetate and glycerol was, however, enhanced by about 40% in the mutant XR strain under these conditions. Interestingly, the effect of altered cosubstrate specificity of *P. stipitis *XR on ethanol yield was not clearly visible in strains that harbored two copies of the respective XR gene and hence consumed xylose about 1.5-fold faster than the corresponding single-copy strains.

In a continuous culture that used a mixed sugar substrate (10 g/L glucose, 10 g/L xylose), the strain harboring a single gene copy for the K270M mutant produced 8% more ethanol (0.40 g/g) and 41% less xylitol than the corresponding control strain. It was suggested from results of metabolic flux analysis that xylose conversion by the K270M mutant took place exclusively via NADH-dependent reaction while the wild-type form of *P. stipitis *XR showed balanced utilization of NADH and NADPH under these conditions (see later). Unfortunately, significant differences in physiological parameters for the native XR strains BP000 (*Y*_EtOH/xylose _= 0.24; *q*_xylose _= 0.06 h^-1^, where *Y *is a yield coefficient and *q *is the specific uptake rate) and TMB3001 (*Y*_EtOH/xylose _= 0.31; *q*_xylose _= 0.145 h^-1 ^[56]) set a limit to the quantitative evaluation of the possible benefit, particularly on *Y*_EtOH/xylose_, originating from the use of the K274R-N276D double mutant of *Ct*XR (this work) compared to the K270M mutant of the *P. stipitis *enzyme [56].

Notwithstanding, if we assume that quantitative information about XR performance under *in vivo *conditions can be gleaned from the results of relevant *in vitro *assays [40,41], the *Ct*XR double mutant is expected to be a much superior catalyst with regard to both coenzyme selectivity and efficiency. Unfortunately, the large preference for NADPH seen with isolated preparations of native *P. stipitis *XR [45,46] is very difficult to reconcile with the suggestion from metabolic flux analysis that a very substantial fraction of xylose (≈ 50%) is consumed by the enzyme *in vivo *via the NADH-dependent pathway [56,60]. Therefore, while further systematic integration of XR protein engineering into the development of novel xylose-fermenting strains of *S. cerevisiae *would seem to be a promising approach, it also requires that the apparent conflict in findings for *in vitro *and *in vivo *experiments be sorted out in future studies.

## Methods

### Strains and plasmids

*Escherichia coli *strain TOP10 (Invitrogen, Carlsbad, CA, USA) was used as bacterial host for subcloning. *Saccharomyces cerevisiae *strain CEN.PK 113-7D (MATαMAL2-8c SUC2) was used for the isolation of yeast genomic DNA and as reference. Recombinant yeast strains were derived from uracil-deficient *S. cerevisiae *strain CEN.PK 113-5D. Plasmids pET11-*Ct*XRWt [61] and pET11-*Ct*XRDm [40] carry the genes encoding native and K274R-N276D double mutant forms of XR from *Candida tenuis *CBS4435, respectively. Plasmid pBTac1 [62] carries the gene encoding XDH from *Galactocandida mastotermitis*. Construction of gene cassettes for expression in *S. cerevisiae *was performed using plasmid pRS416GPD [63]. Yeast integrating vector YIp5 (DSMZ, Braunschweig, Germany) [40] was used for chromosomal insertion of the respective gene cassette.

### Media

Bacterial transformants were selected on Luria-Bertani medium agar plates supplemented with 112 mg/L ampicillin. Prior to transformation, yeast cells were grown in YPD medium. Transformants were selected on yeast synthetic complete media agar plates prepared from Yeast Nitrogen Base (Sigma, St. Louis, MO, USA) that contained Yeast Synthetic Drop-out Medium Supplements (Sigma) lacking uracil. Xylose fermentations in shake-flask and bioreactor cultivations were performed using a defined mineral medium containing vitamins and trace elements [56]. The medium was supplemented with 0.01 g/L ergosterol, 0.42 g/L Tween 80 (dissolved in boiling 96 vol% ethanol), and 100 mM sodium citrate buffer, pH 5.5.

### Construction of yeast integrating vectors

Restriction enzymes were from MBI Fermentas (St. Leon-Roth, Germany) or New England Biolabs (Beverly, MA, USA). *Pfu *DNA polymerase was from Promega (Madison, WI, USA). QIAprep Spin Miniprep Kit from Qiagen (Quiagen GmBH, Hilden, Germany) was used for plasmid preparation, and QIAquick Gel Extraction Kit was used for DNA extraction from agarose. Genomic DNA was isolated with the DNEasy Tissue Kit from Quiagen. Standard techniques of recombinant DNA technology and molecular biology were used.

**Figure 5:**
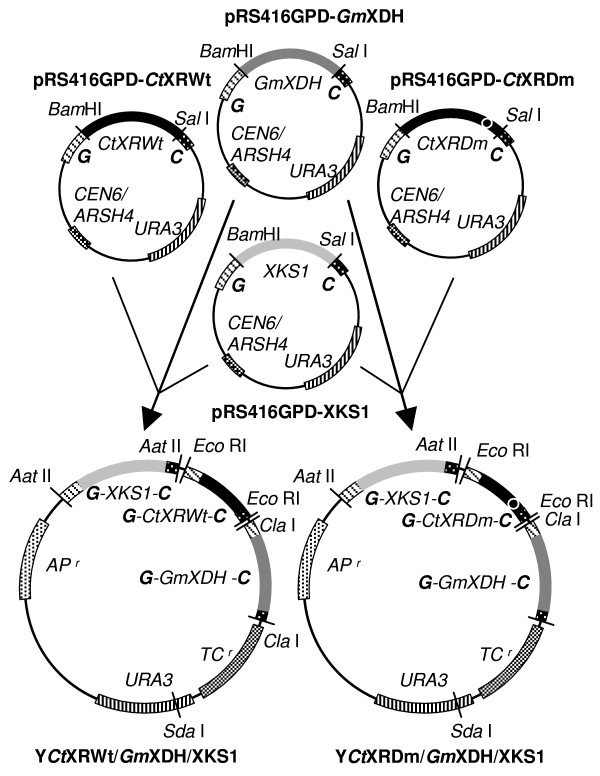
**Cloning strategy employed for construction of plasmids Y*Ct*XRWt/*Gm*XHD/XKS1 and Y*Ct*XRWDm/*Gm*XHD/XKS1**. Further details are given in Table 4 and text. The TDH3 promoter and the CYC1 terminator are labeled G and C, respectively.

In a first step, the promoterless genes for native or K274R-N276D *Ct*XR, *Gm*XDH, and the endogenous yeast XK1 were amplified from pET11-*Ct*XRWt or pET11-*Ct*XRDm, pBTac1, and genomic *S. cerevisiae *DNA, respectively. Polymerase chain reactions were performed using forward and reverse oligonucleotide primers whose 5'-ends contained a *Bam*HI and *Sal*I restriction site, respectively (see Table 4). Amplification products were digested with *Bam*HI and *Sal*I and inserted into the multiple cloning site of pRS416GPD, situated between the TDH3, formerly glyceraldehyde 3-phosphate dehydrogenase (GPD) promoter and the cytochrome-c-oxidase (CYC1) terminator. Gene cassettes were constructed where each of the target genes (XRWt, XRDm, XDH, and XK) was integrated separately between a TDH3 promoter and CYC1 terminator. Correct insertion was verified by sequencing. In a second step, the gene cassettes were amplified by PCR using oligonucleotide primers containing restriction sites at their respective 5'-end that are unique for YIp5 (see Table 4, Figure 5). The XK gene cassette was cloned into the *Aat*II site, resulting in vector YXKS1. Third, the XDH gene cassette was inserted into the *Cla*I site of YXKS1, resulting in Y*Gm*XDH/XKS1. Fourth and finally, the gene cassette for either XRWt or XRDm was cloned into the *EcoR*I site of Y*Gm*XDH/XKS1, resulting in Y*Ct*XRWt/*Gm*XDH/XKS1 and Y*Ct*XRDm/*Gm*XDH/XKS1. Correct orientation of the inserted gene cassettes was verified after each step of integration using PCR screening with a pair of oligonucleotide primers matching a sequence upstream of the cloning site in the target vector and a sequence of the inserted gene.

**Table 4:** Cloning strategy for the construction of yeast integrating plasmids Y*Ct*XRWt/*Gm*XDH/XKS1 and Y*Ct*XRDm/*Gm*XDH/XKS1. The shown primer sets were used to amplify the target sequences from the template plasmids. The amplification products were cloned into the corresponding restriction sites of the target plasmid.

	**Target sequence**	**Template plasmid**	**Primers (restriction sites underlined)**	**Target plasmid**	**Restriction sites**	**Resulting plasmid**
1	*Ct*XRWt gene	pET11-*Ct*XRWt	Fwd: 5'-GGTGGTGGATCCATGAG CGCAAGTATCCGAGAC-3'	pRS416GPD	*BamH*I *Sal*I	pRS416GPD-*Ct*XRWt
	*Ct*XRDm gene	pET11-*Ct*XRDm	Rev: 5'CTAGTGGGTCGACTTAAAC GAAGATTGGAATGTTGTC-3'	pRS416GPD	*BamH*I *Sal*I	pRS416GPD-*Ct*XRDm
	*Gm*XDH gene	pBTac1	Fwd: 5'-GGTGGTGGATCCATGTCT ACTCCTGAAAACTTATCT-3'	pRS416GPD	*BamH*I *Sal*I	pRS416GPD-*Gm*XDH
			Rev: 5'-CTAGTGGGTCGACTTAC TCAGGGCCGTTAATGATG-3'			
	XKS1 gene	Genomic *S. cerevisiae *DNA	Fwd: 5'-GGTGGTGGATCCATGTTG TGTTCAGTAATTCAGAGA-3'	pRS416GPD	*BamH*I *Sal*I	pRS416GPD-XKS1
			Rev: 5'-GGTGGTGTCGACTTAGAT GAGAGTCTTTTCCAGTTC-3'			
2	Gene cassette^a ^XKS1	pRS416GPD-XKS1	Fwd: 5'-CATGGTGACGTCAGTTTATC ATTATCAATACTCGCCATTTC-3'	YiP5	*Aat*II	YXKS1
			Rev: 5'-GGTGGTGACGTCGGCCGCA AATTAAAGCCTTCG-3'			
3	Gene cassette *Gm*XDH	pRS416GPD-*Gm*XDH	Fwd: 5'-CATGGTATCGATAGTTTATC ATTATCAATACTCGCCATTTC-3'	YXKS1	*Cla*I	Y*Gm*XDH/XKS1
			Rev: 5'-GGTGGTATCGATGGCCGCA AATTAAAGCCTTCG-3'			
4	Gene cassette *Ct*XRWt	pRS416GPD-*Ct*XRWt	Fwd: 5'-GGTGGTGAATTCAGTTTATC ATTATCAATACTCGCCATTTC-3'	Y*Gm*XDH/XKS1	*EcoR*I	Y*Ct*XRWt/*Gm*XHD/XKS1
	Gene cassette *Ct*XRDm	pRS416GPD-*Ct*XRDm	Rev: 5'-GGTGGTGAATTCGGCCGCA AATTAAAGCCTTCG-3'	Y*Gm*XDH/XKS1	*EcoR*I	Y*Ct*XRDm/*Gm*XHD/XKS1

^a ^Gene cassettes contain *S. cerevisiae *TDH3 promoter (labeled GPD in the Table) – target gene – *S. cerevisiae *CYC1 terminator

### Transformation

Transformation of plasmids into Top10 competent cells was done by electroporation. Yeast integrating plasmids Y*Ct*XRWt/*Gm*XDH/XKS1 and Y*Ct*XRDm/*Gm*XDH/XKS1 were cleaved by *Sda*I within the URA3 gene. The linearized vectors were transformed into *S. cerevisiae *CEN.PK 113-5D using the lithium acetate method [64], resulting in the strains BP000 and BP10001 that express the gene encoding wild-type *Ct*XR and the K274R-N276D double mutant thereof, respectively. Selected yeast strains were stored in 15% glycerol at -80°C.

### Fermentation of xylose under oxygen-limited culture conditions in shake flasks

Oxygen limitation during batch conversion of xylose by BP000 and BP10001 was achieved using 300-mL baffled shake flasks that were tightly closed with rubber stoppers. Two glass tubes were inserted in the stopper, one with a valve for purging with nitrogen and another containing a narrow slit at its closed far end which served as gas outlet. A magnetic stirrer bar (3 cm in diameter) was added to each shake flask. A fluorescence-based fiber-optic sensor (PreSens GmbH, Regensburg, Germany) was used to measure the concentration of dissolved O_2 _in the medium each time when a sample was taken. The O_2 _concentration never exceeded a value of 20 μM.

Yeast cells were grown overnight at 30°C and 110 rpm using a defined mineral medium that contained 20 g/L glucose. They were harvested by centrifugation (10 min; 4400 *g*) and after washing twice with 0.9% NaCl used for inoculation, to give a final optical density of ≈ 4. The working volume of each shake flask was 280 mL, and mineral medium containing 20 g/L xylose was used. The concentration of xylose at the start of the fermentation was increased in some cases to about 21 g/L as result of evaporation during the sterilisation. Note that reported data is always from measurements of the actual sugar concentrations. Shake flasks were purged with N_2 _containing less than 5 ppm O_2 _for 15 minutes before and 5 minutes after the inoculation. Further incubation of the sealed flasks was carried out at 30°C and 100 rpm using a Sartorius incubator. Care was taken that during withdrawing a sample (≈ 3 mL) from the shake flask, the biomass was homogeneously suspended. This was achieved by magnetic stirring and done under nitrogen purging. Work-up of samples and analytical procedures are described in a separate section, Analyses. Xylose fermentations were done in triplicate for each strain, and the results show mean values and the corresponding S.D. (Table 3, Figure 3)

### Fermentation of xylose in anaerobic bioreactor cultivations

A Braun Biostat C bioreactor equipped with two six-bladed disc impellers was used. The bioreactor had a working volume of 4 L. The ratio of impeller to reactor diameter was 0.4. Fermentations were carried out under conditions exactly comparable to the ones used for shake-flask experiments. The stirrer speed was set to a constant value of 200 rpm. The reactor was sparged with N_2 _at a flow rate of 0.5 L/min. The pH was controlled at a value of 5.0 through automatic addition of 1 M NaOH.

### Carbon balance and ethanol evaporation

Carbon balances for xylose fermentation in shake flasks are based on the assumption that 1 mole of CO_2 _is formed per mole of ethanol and acetate. For carbon balances for fermentations in the bioreactor, CO_2 _was calculated from the off gas analysis. Due to sparging with N_2_, ethanol is evaporated from the bioreactor. The rate of ethanol evaporation was determined at a N_2 _flow rate of 0.5 L/min, measuring by HPLC the decrease in the ethanol concentration as a function of time. Mineral medium lacking biomass was supplemented with 3 concentrations of ethanol between 1 and 4.5 g/L. Time-dependent loss of ethanol from this mixture could be described by a first-order decay function with a rate constant of 4.4 10^-3 ^h^-1^. Reported values for the ethanol produced include the calculated evaporated alcohol.

### Analyses

#### Cell growth and cell dry weight

Optical density at 600 nm (OD_600_) was used to monitor cell growth. Cell dry weight (CDW) was determined by filtering 50 mL sample through a 0.45-μm cellulose acetate membrane filter (Sartorius type 111, 47 mm diameter; Satorius, Göttingen, Germany) that had been dried (110°C, 12 h) and weighed prior to use. After two washes of the filter cake with deionized water, the filter was dried overnight at 105°C and then weighed. The relationship between OD_600 _and CDW was carefully calibrated.

#### Off gas analysis

The concentrations of CO_2 _and O_2 _in the bioreactor off gas were measured with an IN1313 acoustic gas analyzer (Innova AirTech Instruments, Ballerup, DK) according to instructions by the instrument supplier.

#### Extracellular products

Samples taken from shake flasks or the bioreactor were filtered with a Satorius Minisart RC4 filter and unless used immediately, stored at -20°C. The product distribution resulting from xylose fermentation was analyzed by HPLC. A Merck-Hitachi LaChrome HPLC System equipped with an Aminex HPX-87H (Biorad, Richmond, CA, USA) column, a Merck-Hitachi LaChrome L-7250 autosampler and a Merck L-7490 RI detector was used. The system was operated at 65°C, using a flow rate of 0.6 mL/h for the eluent (5 mM sulfuric acid). Under these conditions, glucose, xylose, xylitol, glycerol, ethanol and acetate could be analyzed quantitatively.

#### Determination of intracellular enzyme activities

Yeast cells were grown in shake flasks under aerobic conditions at 30°C using a defined mineral medium that contained 20 g/L glucose and was optionally supplemented with 20 g/L xylose. Agitation was at 140 rpm. They were harvested in the mid-exponential growth phase using centrifugation (10 min; 4400 *g*). After washing twice with saline, the cell material was treated with the lysis reagent Y-PER (Pierce, Rockford, IL, USA) according to instructions of the supplier or disrupted in a French Press (SLM-Aminco French Press Mini Cell; 19000 psi internal cell pressure, two passages). The crude cell extract obtained by either of the two methods was used for determination of total protein, employing the Roti-Quant protein assay (Carl Roth GmbH, Karlsruhe, Germany) referenced against BSA fraction 5, and for enzyme activity measurements. Standard spectrophotometric assays for XR, XDH, and XK activity were described previously. Briefly, initial rates of XR-catalyzed reduction of xylose (700 mM) were measured, unless indicated otherwise, in the presence of 350 μM NADH or NADPH [40]. The XDH activity was determined using 150 mM xylitol and 2 mM NAD^+ ^[65]. The continuous coupled enzymatic assay for XK activity contained 5 mM ATP and 4.3 mM D-xylulose [66]. Relevant controls were recorded in all cases, and reported values are corrected for the blank readings. When measuring XK activity, it was particularly important to take into account the blank resulting from the XDH-catalyzed reduction of D-xylulose by the NADH present in the assay mixture. A Beckman Coulter DU 800 UV/Vis spectrophotometer was used to monitor enzymatic rates of formation (XDH) or depletion of NADH (XR, XDH) and NADPH (XR) at 340 nm.

## Competing interests

The author(s) declare that they have no competing interests.

## Authors' contributions

All authors have read and approved the final manuscript. BP and BN designed research; BP performed experiments and analyzed data; BP and BN wrote the paper.
